# Assessing the technical capability of a room- and a gantry-mounted kV imaging device for intra-fractional fluoroscopy during stereotactic lung radiotherapy at a C-arm linear accelerator

**DOI:** 10.1016/j.phro.2026.100988

**Published:** 2026-05-02

**Authors:** Hannah Jungreuthmayer, Barbara Knäusl, Julius Arnold, Martin Buschmann, Andreas Renner, Maximilian Schmid, Dietmar Georg, Wolfgang Lechner

**Affiliations:** a Medical University of Vienna, Department of Radiation Oncology, Vienna, Austria; b Christian Doppler Laboratory for Image and Knowledge Driven Precision Radiation Oncology, Medical University of Vienna, Vienna, Austria

**Keywords:** Stereotactic radiotherapy, Lung tumor, Fluoroscopy, kV imaging

## Abstract

**Background and Purpose:**

Monitoring lung tumors during stereotactic radiotherapy is important for managing breathing motion, yet the high-performance imaging modules on C-arm linear accelerators are mainly used for patient positioning. This study benchmarked a room-mounted against a gantry-mounted kilovolt (kV) imaging device to assess their potential for lung tumor fluoroscopy.

**Materials and Methods:**

The investigated systems were the ExacTrac Dynamic (ETD, Brainlab, Germany) and the X-ray Volumetric Imaging (XVI, Elekta, Sweden). Contrast-to-Noise Ratio (CNR) and entrance air kerma were measured for different imaging settings — tube voltage (70 kV to 130 kV), current (10 mA to 320 mA), exposure time (100 ms and 40 ms) — using two anthropomorphic thorax phantoms housing three tumors. Additionally, CNR change as a function of time, achievable fluoroscopy imaging duration based on X-ray generator and anode heat and X-ray tube cool-down were evaluated.

**Results:**

CNRs agreed within 15% for the two systems and changed less than 2% over the investigated fluoroscopy durations. Using default imaging settings and considering the average clinical duration of stereotactic lung treatments, entrance air kermas were 44 mGy (room-mounted) and 41 mGy (gantry-mounted). Achievable fluoroscopy durations with default settings were 87 s and 1350 s, respectively. Anode cool-down times were 125 min (room-mounted) and 215 min (gantry-mounted).

**Conclusion:**

The performance metrics of the room-mounted imaging system aligned with those of the gantry-mounted device with regard to CNR and air kerma. The room-mounted system showed a preferable cool-down behavior. However, adaptation of imaging settings is necessary to increase the fluoroscopy duration and avoid overheating.

## Highlights

• Room- and gantry-mounted systems were suitable for intra-fractional kV fluoroscopy
• Phantom tumor contrast-to-noise ratios agreed within 15% for both systems.
• Calculated imaging entrance air kerma was <3% of the treatment related skin dose.
• Cool-down times were 125 min (room-mounted) and 215 min (gantry-mounted).
• Optimization of imaging settings can extend the feasible fluoroscopy duration.

## Graphical abstract

**Figure:**
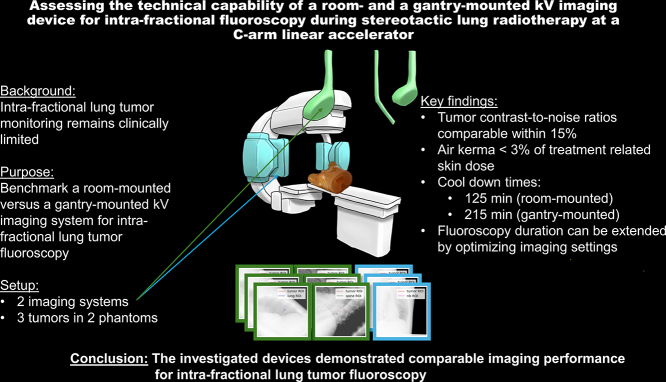

## 1 Introduction

Stereotactic radiotherapy is frequently applied for treating lung tumors, due to the ability to target tumor tissue and spare functional lung tissue [1]. However, intra-fractional anatomical changes, like breathing motion, make this challenging [2]. Most treatment concepts take lung tumor motion into account by using an internal target volume margin, accounting for these variations [3], [4]. This promotes target coverage at the cost of larger irradiated volumes [5], [6]. Techniques such as gating or tracking can potentially reduce the margins but require high-frequency real-time tumor motion monitoring, considering the short breathing period of around 4 s [2], [7], [8].

Today, dedicated machines with fully integrated tracking/gating systems exist but are mostly based on markers and scarcely available [9], [10], [11], [12], [13], [14], [15], [16], [17]. State-of-the-art C-arm linear accelerators (LINACs) are equipped with kilovoltage (kV) X-ray imaging systems, potentially applicable for clinical tumor motion monitoring [12], [18]. Intra-fractional X-ray imaging, independent of the tracking system, leads to an additional dose burden, especially to the skin, necessitating evaluation and system-specific optimization of imaging settings [19], [20].

Marker-based X-ray motion monitoring has already been applied at C-arm LINACs, either via kV imaging alone or in combination with megavoltage or optical imaging [21], [22], [23]. However, marker implantation poses additional risks (e.g., pneumothorax) and is constrained by issues like marker migration [22], [24]. Markerless monitoring offers an alternative for lung tumors with high density differences between normal and malignant tissue. The utility of gantry-mounted kV imaging modules — whether dedicated devices or customized solutions — has been established in several studies, showing promising accuracy for tumor monitoring in both phantom and clinical settings [15], [25], [26], [27], [28], [29], [30], [31], [32], [33], [34]. Although technically feasible for a decade, these were not widely adopted for clinical implementation. Major challenges are related to monoscopic vision [35] and dense structure obstruction at certain gantry angles [26], but also latencies [36], tumor localization methods [27] and synchronization with other systems [37]. Insights on clinical (markerless) intra-fractional lung tumor motion monitoring at C-arm LINACs, in particular with stereoscopic room-mounted imaging systems, remains limited.

This proof-of-principle study provides a device-specific characterization of a stereoscopic room-mounted imaging system for continuous markerless intra-fractional fluoroscopy during stereotactic lung tumor radiotherapy at a C-arm LINAC, benchmarked against a gantry-mounted system. The systematic characterization was performed by investigating the effect of various imaging settings on tumor Contrast-to-Noise Ratio (CNR) and entrance air kerma in anthropomorphic phantoms. CNR changes as a function of time, achievable imaging duration based on the X-ray generator and anode heat and cool-down behavior, were assessed as preparation for future clinical implementation.

## 2 Materials and methods

### 2.1 Imaging devices, phantoms and dosimeter

The investigated imaging systems were the room-mounted ExacTrac Dynamic (ETD) v2.0 (Brainlab SE, Munich, Germany) and the gantry-mounted X-ray Volumetric Imaging (XVI) v5.0 (Elekta AB, Stockholm, Sweden), both integrated with an Elekta Versa LINAC. ETD was equipped with a water cooling system and a novel prototype software, not yet clinically available. The prototype software enabled monoscopic and stereoscopic fluoroscopy (up to 2.56 Hz at 100 ms exposure time) and synchronized high-frequency surface recordings using the unaltered clinical hardware [38]. The clinical XVI supports monoscopic fluoroscopy at 5 Hz, using the clinical MotionView tool and the S20 collimator without a filter (clinical standard [39]) and has been used previously for various tasks related to intra-fractional lung tumor motion monitoring [35], [40], [41]. Both devices featured 16-bit pixel depth.

**Fig. 1:**
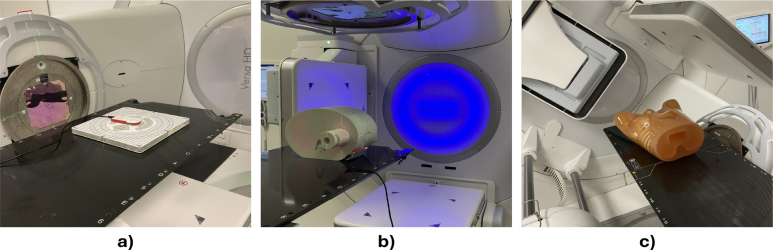
Exemplary phantom setups (a) Imaging the NORMI RAD/FLU phantom with the XVI system; (b) Measuring ETD doses on the CIRS phantom surface; (c) Imaging the LUNGMAN phantom with the XVI system.

The CIRS Dynamic thorax phantom (CIRS, Norfolk, USA) with a spherical tumor insert (20 mm), the LUNGMAN thorax phantom (Kyoto Kagaku, Kyoto, Japan) with two spherical tumor inserts (*tumor 1*: right lower lobe, 10 mm, 100HU; *tumor 2*: left lower lobe, 12 mm, 100 HU) and the NORMI RAD/FLU (PTW, Freiburg, Germany) were used for imaging (Fig. 1). For dose measurements the NOMEX Multimeter (PTW, Freiburg, Germany) was utilized.

Additional technical specifications are provided in Supplementary Section A [42], [43], [44].

### 2.2 Measurement setup

Images of the anthropomorphic phantoms were acquired with the tumor placed in the isocenter.

For monoscopic ETD image acquisition with the CIRS and the NORMI RAD/FLU phantom the patient couch was rotated to 45°to provide a similar imaging geometry as XVI. Two stands held the NORMI RAD/FLU phantom perpendicular to the beam. For the LUNGMAN phantom stereoscopic ETD images were acquired without table rotation to simulate real patient cases.

XVI image acquisition with the CIRS phantom was performed with the gantry rotated to 45°to ensure comparability between the imaging systems. For imaging the NORMI RAD/FLU phantom the gantry was rotated to 270°(Fig. 1a). For imaging LUNGMAN *tumor 1*, the gantry was rotated to 135°, comparable to the ETD imaging angle. For LUNGMAN *tumor 2*, in addition to 45°, three other gantry angles (0°, 90°, 135°) were tested for one imaging setting, representing a selection of angles typically included during volumetric modulated arc therapy (VMAT).

Dose measurements were performed without table rotation for both devices and with a gantry angle of 45°for XVI.

**Table 1:** Characteristics and investigated imaging settings for both devices. The default settings for lung image acquisition are written in **bold**.

Device	Characteristics	Frequency (Hz)	Exposure time (ms)	Tube voltage (kV)	Tube current (mA)
ETD prototype	room-mounted stereoscopic	2.56	**100**	70, 80, 90, 100, **110**, 120, 130	40, 50, 60, 80, 100, 120, **160**, 200, 250, 320

XVI	gantry-mounted monoscopic	5	**40**	70, 100, **120**	10, 12, 16, 20, **25**, 32, 40, 50, 64, 80, 100, 125, 160, 200, 250

### 2.3 Image quality

For all imaging settings (Table 1), CNR was assessed in the CIRS phantom (initial evaluation of imaging parameters and possibility for cross-institutional replicability) and the LUNGMAN phantom (more realistic patient cases). The employed tube voltages and currents encompassed the full operational range for XVI and representative clinical settings for ETD. Considering the different imaging geometries of the two systems these ranges were not comparable.

Eq. (1) was used to calculate the CNR between the tumor and a tissue region of interest (ROI) with similar background (ROI dimensions depended on the tumor size: CIRS: 1 cm × 1 cm, LUNGMAN: 0.5 cm × 0.5 cm).

(1) $$CNR=\frac{I_{Tumor}-I_{Tissue}}{\sigma_{Tissue}}$$

**Fig. 2:**
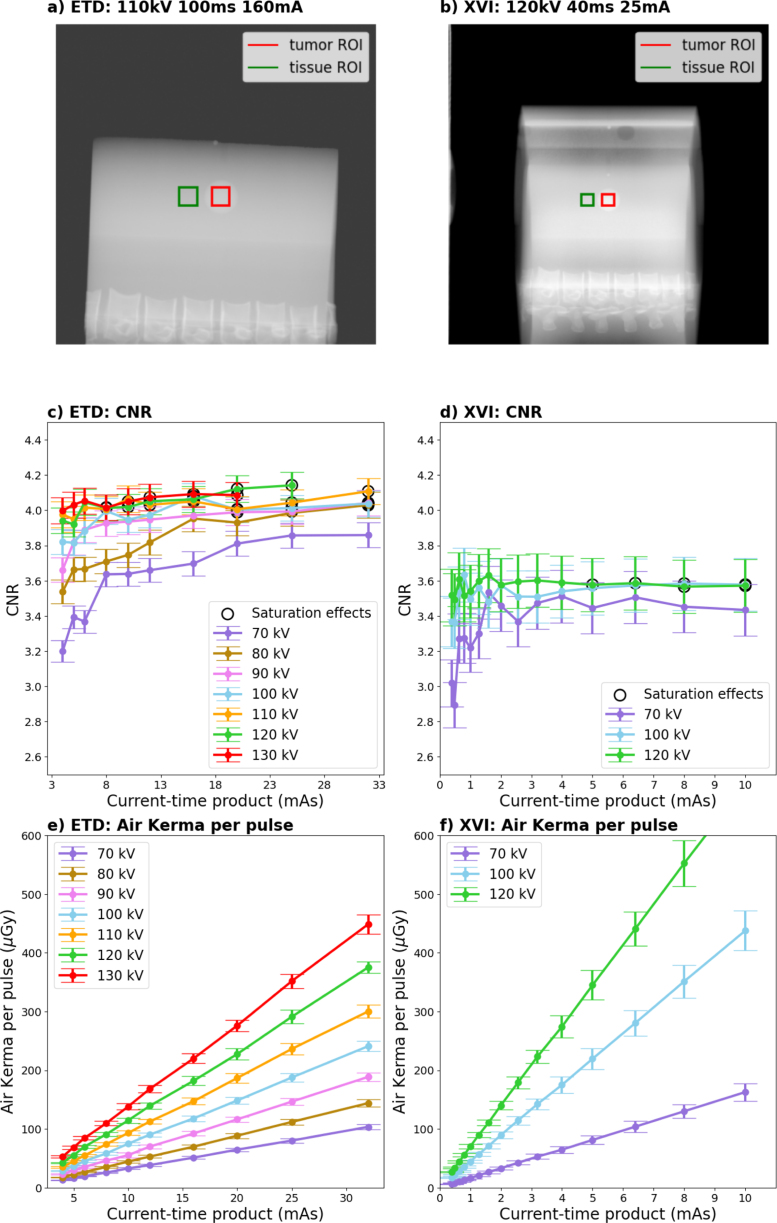
(a) and (b) show an ETD prototype and an XVI CIRS phantom image acquired with the respective default setting. ROIs in the tumor and surrounding tissue are indicated. (c) and (d) show the CNR between tumor and surrounding tissue ROIs as a function of the current-time product for different tube voltages and both imaging devices. Data points marked with a black circle contained saturation effects in the periphery. (e) and (f) show the corresponding entrance air kerma.

$I_{Tumor}$, $I_{Tissue}$ and $\sigma_{Tissue}$ are the average pixel values and standard deviation in the tumor and tissue ROIs. The uncertainty of CNR was calculated by applying Gaussian error propagation. In case of saturation effects (maximum intensity) in the tumor region, the CNR was not evaluated. For CNR assessment, unfiltered raw images were used. However, for visualization, the XVI CIRS phantom images (Fig. 2b, Supplementary Figure S1b and d, S2b and d) were contrast enhanced using Contrast Limited Adaptive Histogram Equalization (CLAHE) (python module openCV version 4.11.0.86: clipLimit = 2, tileGridSize = [6 × 6]), while all other images remained unfiltered. CNR change during fluoroscopy was investigated by imaging the static NORMI RAD/FLU and the CIRS phantom with and without tumor motion. Default imaging settings were used for data acquisition and the CNR change between two ROIs as a function of time was evaluated. For the NORMI RAD/FLU phantom the ROIs were placed in a low contrast region and the surrounding background (ROI dimensions NORMI RAD/FLU: 0.5 cm × 0.5 cm), while for the static CIRS phantom the ROIs were in the tumor and the surrounding tissue. For the moving CIRS phantom, the investigated tumor motion curve was $\cos^{4}$ with a superior-inferior motion of 12 mm and a breathing period of 3.6 s [2]. To locate the tumor position for ROI placement and CNR calculation, a line profile through the tumor was drawn and fitted with a Gaussian (scipy 1.16.1). The tissue ROI was static and outside of the tumor motion range. Three consecutive fluoroscopy sequences with the maximum frame number were acquired with ETD. Only a single sequence was recorded with XVI, due to its higher frame capacity (see Sections 2.5, 3). A linear regression of CNR over time was performed using the python module statsmodels.api (version 0.14.4), to determine the slope and its uncertainty. Outliers (z-score ≥ 3) were excluded from the fit. The significance of the slope was estimated using a two-tailed t-test (significance level p=0.05).

### 2.4 Imaging entrance air kerma assessment

Single frame entrance air kerma was measured with the NOMEX Multimeter placed roughly in the beam’s central axis, on the beam entrance side of the CIRS surface [45], [46]. For ETD, stereoscopic images were acquired with the NOMEX Multimeter placed in the entrance path of one of the beams. Table 1 lists the investigated imaging settings. All measurements were repeated three times, each on a different day, yielding a mean and standard deviation. All uncertainties taken into account are listed in Supplementary Table S1 [47].

### 2.5 Feasible fluoroscopy duration and anode cool-down

The feasible frame number with the ETD prototype was limited by three independent factors: (1) a software-imposed 80% anode heat limit (maximum anode heat content 635 kJ [48]), (2) a limit of 1200 mA s per fluoroscopy sequence to prevent generator errors, and (3) manufacturer-defined tube load limits <100 kW (depending on acquisition frequency, exposure time and number of exposures in series [48]). The maximum possible frame number per fluoroscopy sequence was evaluated for different imaging settings, with a fixed exposure time of 100 ms and a frequency of 2.56 Hz. The feasible number of consecutive sequences using the maximum frame number was tested for non-exhaustive combinations of the following tube voltage and current settings: 90 kV, 100 kV, 110 kV, 120 kV, 130 kV combined with 40 mA, 50 mA, 60 mA, 80 mA, 100 mA, 120 mA, 160 mA, 200 mA.

The XVI software allowed a maximum of 3300 frames per sequence irrespective of the imaging setting and the anode heat. However, there is a documented XVI generator limit of 800 kJ [39]. This limit was confirmed by acquiring several consecutive fluoroscopy sequences with the default imaging setting and the maximum frame number. For XVI the total feasible frame number with specific settings was calculated based on the documented generator limit.

Anode cooling curves were recorded for both devices after reaching a fluoroscopy limit. For ETD and XVI, 90 kV, 100 ms, 80 mA and 120 kV, 40 ms, 25 mA were used, respectively (chosen as example settings). ETD cooling data was extracted from system logs, which included both the generator-reported cooling curve and a prototype software-generated approximation used for calculations. XVI cooling was tracked by reading the heat units manually every five minutes.

### 2.6 Comparison to clinical treatment plans of stereotactic lung tumor patients

An average beam-on time was calculated based on LINAC log-files of 23 stereotactic lung treatment plans (prescription: Planning Target Volume (PTV) D${}_{95\text{\%}}$ 28 Gy to 60 Gy; near maximum dose: ≤ 154%; fraction numbers: 1, 3, 5 and 8), which were normalized to the duration of an 11 Gy fraction (median fraction dose). This average beam-on time was used as benchmark for the required fluoroscopy durations (Section 2.5). As ETD stereoscopic imaging was restricted to cardinal angles and nearby positions, the number of gantry angles facilitating stereoscopic imaging during treatment was calculated based on the same data. Assuming static gantry conditions and continuous acquisition at default imaging settings, a coarse estimate for the total entrance air kerma during an average treatment was calculated by multiplication of the single frame entrance air kerma with the necessary frames (uncertainties included single frame measurement uncertainties and treatment duration variation). For rough comparison, the average treatment skin doses (doses absorbed by the skin) of these 23 plans were calculated within the intersection of a 2 mm thick inward margin from the external contour and the 10% isodose volume using the treatment planning system RayStation 12 A (RaySearch, Stockholm, Sweden), assuming similar skin dose uncertainties than observed by Court et al. [49]. Resulting skin doses were normalized to 11 Gy/fraction (fx) and reported as the mean ± standard deviation averaging over all patients.

## 3 Results

For the CIRS phantom (Fig. 2 and S1), the CNR varied between 3.2 ± 0.07 and 4.14 ± 0.08 for ETD and between 2.9 ± 0.2 and 3.6 ± 0.2 for XVI, being comparable within 15% across the evaluated range. Both CNR curves showed a flattening close to their maximum value, with increasing saturation effects (Fig. 2c and d). Using the default imaging settings, the CIRS CNR and entrance air kerma per frame (Fig. 2e and f) were 4.05 ± 0.08 and 147 ± 6 µGy for ETD and 3.5 ± 0.2 and 70 ± 4 µGy for XVI.

**Fig. 3:**
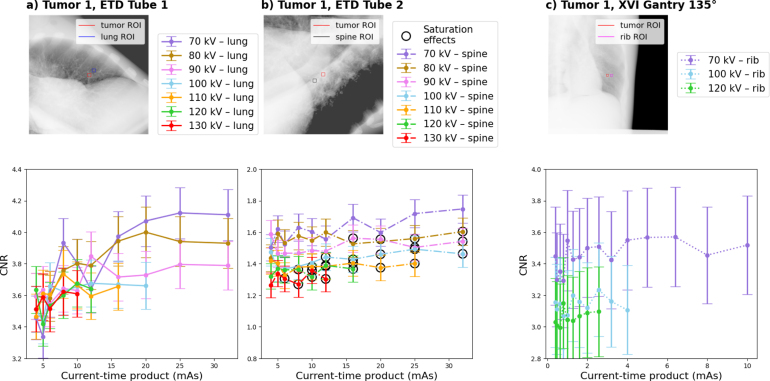
(a) and (b) show ETD prototype images for LUNGMAN *tumor 1* with both tubes. (c) shows the XVI image with a gantry angle of 135°. Images were acquired with the respective default setting. ROIs are indicated in the tumor and surrounding tissue. Below these images the respective CNR between tumor and surrounding tissue ROI as a function of the current-time product for different tube voltages and both imaging devices is shown. Data points marked with a black circle contained saturation effects in the periphery.

**Fig. 4:**
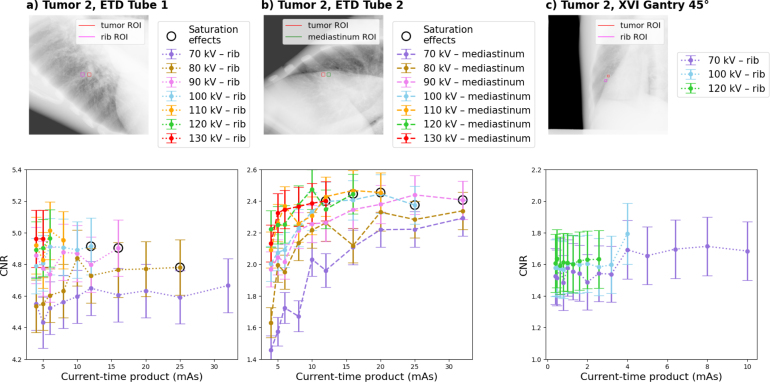
(a) and (b) show ETD prototype images for LUNGMAN *tumor 2* with both tubes. (c) shows the XVI image with a gantry angle of 45°. (b) and (c) were acquired with the respective default setting, while (a) was acquired with 110 kV and 8 mA s, due saturation effects in the tumor region. ROIs are indicated in the tumor and surrounding tissues. Below these images the respective CNR between tumor and surrounding tissue ROI as a function of the current-time product for different tube voltages and both imaging devices is shown. Data points marked with a black circle contained saturation effects in the periphery.

Employing the default lung imaging settings, LUNGMAN *tumor 1* CNRs were 3.7 ± 0.2 for ETD tube 1, 1.40 ± 0.09 for ETD tube 2 and 3.0 ± 0.3 for XVI (Fig. 3). For LUNGMAN *tumor 2*, CNRs were 2.5 ± 0.2 for ETD tube 2 and 1.6 ± 0.2 for XVI (Fig. 4). For ETD tube 1, CNR could not be evaluated due to saturation effects in the tumor region. For LUNGMAN *tumor 2*, the default XVI imaging setting led to a varying CNR for different gantry angles (which required the consideration of different background tissues): 1.6 ± 0.2 at 135°(bronchioles), 2.6 ± 0.3 at 0°(mediastinum) and 1.2 ± 0.2 at 90°(rib) (Supplementary Figure S3).

Except for the XVI fluoroscopy sequence with the NORMI RAD/FLU phantom with a CNR slope of (8 ± 2) $\times 10^{-5}$ (p = 5.5$\times 10^{-5}$), corresponding to an average CNR change over fluoroscopy duration of less than 2%, none of the calculated CNR slopes were statistically significant (Supplementary Figure S4 and S5).

**Fig. 5:**
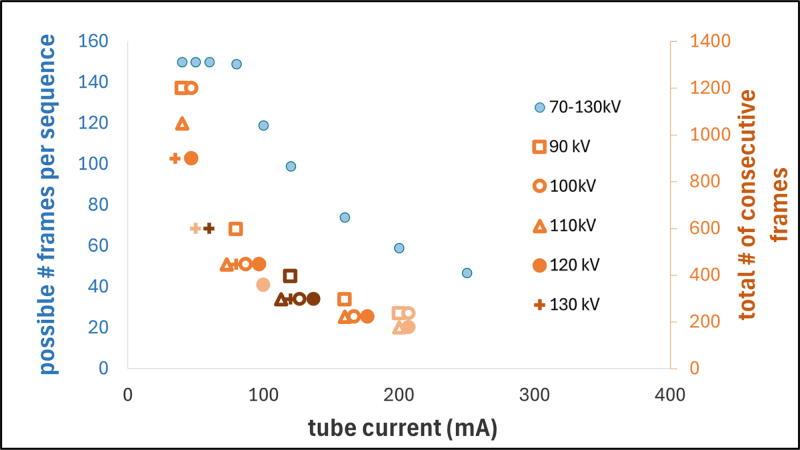
ETD prototype limitations in frame number for fluoroscopy. The primary axis (blue) shows the maximum frame number per fluoroscopy sequence as a function of tube current. The total frame number is also indicated for some settings on the secondary axis (orange). Orange data points with the same tube current are shifted slightly in x-direction due to overlapping with other data points but share the same shade of orange.

For ETD the limiting factors for the possible frame number per fluoroscopy sequence and the total frame number during consecutive sequences were the total tube current-time product and the anode heat, respectively (Fig. 5). For the default imaging setting, 222 frames were feasible with the ETD in total, corresponding to a feasible imaging duration of 87 s. The highest calculated tube energy during these ETD experiments was 480 kJ per tube (100 kV, 40 mA, 1200 frames), representing 76% of the maximum anode heat content.

With the default XVI imaging setting two full sequences of 3299 frames and a third truncated at 151 frames (due to overheating) were feasible (imaging duration of 1350 s). Inherent XVI offset calibration processes between sequences allowed partial cooling. The total required tube energy was 810 kJ, confirming the documented XVI generator limit.

The ETD X-ray generator reported 0 heat units after 47 min, while the prototype approximation reported stabilization at ∼8% residual heat after 125 min. XVI required 215 min to cool down to 0 heat units; no imaging was possible during the first 34 min post-limit (Supplementary Figure S6).

The average beam-on time for stereotactic lung treatment plans normalized to 11 Gy/fx was 116 ± 41 s, requiring 297 ± 105 and 580 ± 205 frames for a frame rate of 2.56 Hz (ETD) and 5 Hz (XVI). Entrance air kermas per average beam-on time were 44 ± 16 mGy for ETD and 41 ± 15 mGy for XVI. On average 5 cardinal gantry angles (stereoscopic imaging positions) were passed during the whole treatment, with an average of 2.1 per arc using standard beam arrangements. The corresponding average skin dose delivered by an 11 Gy/fx treatment was 2.0 ± 0.4 Gy/fx.

## 4 Discussion

This proof-of-principle study benchmarked the technical performance of a room-mounted against a gantry-mounted kV imaging system, both integrated at a C-arm LINAC, evaluating suitability for continuous intra-fractional fluoroscopy during stereotactic lung radiotherapy. CNRs agreed within 15% and air kermas aligned, considering default imaging settings and average beam-on times. Cool-down behavior was preferable for the room-mounted system. A statistically significant CNR change over fluoroscopy duration was only observed in one out of four cases, possibly due to ghosting effects.

Using default settings, fluoroscopy entrance air kermas per average treatment time remained below 3% of the average treatment skin dose and below the threshold for deterministic effects of 2 Gy [50]. Therefore, daily fluoroscopic imaging adds a similar skin imaging dose as a daily cone-beam computed tomography scan [51], [52]. This is a coarse estimate, as for VMAT-based dose delivery, gantry-mounted systems spread the imaging dose over a larger area, reducing localized dose [20]. Furthermore, entrance doses also depend on the patient geometry. For a more accurate comparison of the imaging to the treatment dose, the measured air-kerma would need to be converted to absorbed dose to water. This was omitted due to lacking beam quality correction factors of the employed detector.

The ETD prototype’s feasible frame number was limited by three independent factors (Section 2.5). Approximation of the cool-down curve in the prototype assumed residual heat, impacting consecutive fluoroscopy sequences. Although ETD fluoroscopy durations achievable with the default imaging setting were shorter than the average treatment duration, it was possible to maintain CNR (non-significant decrease with p<0.05), while reducing the imaging dose and extending the fluoroscopy duration. Exemplarily, reducing the tube current to 8 mA s also led to a CNR of 3.7 ± 0.2 for LUNGMAN *tumor 1* using ETD Tube 1, while extending the fluoroscopy duration to 175 s and reducing the entrance air kerma per average beam-on time to 22 ± 8 mGy. For XVI, fluoroscopy stopped automatically once the tube energy limit was reached, temporarily preventing further imaging. Although its cool-down time exceeded that of ETD, it is unlikely in practice since treatments are typically shorter than the maximum XVI fluoroscopy duration.

In a potential clinical workflow employing these systems for automated tumor tracking, CNR degradation by high-density structures or gantry blockage of one stereoscopic tube, might motivate a reconsideration of clinical VMAT angles or couch configurations. However, studies with gantry-mounted systems showed that monoscopic image information might be sufficient for tumor localization [18], [21], [22], [23], [53], [54]. Furthermore, correlating X-ray imaging with surface information could bridge fluoroscopy gaps, reduce the required fluoroscopy frequency and associated dose, while extending the feasible imaging duration for longer treatments [23], [55], [56]. Combining surface recordings with the investigated fluoroscopy systems will be investigated in future studies.

This study was conducted using two anthropomorphic thorax phantoms, one for systematic evaluation and potential cross-institutional replicability and one for mimicking the patient’s anatomy. Although these phantoms do not provide a universal substitute due to the anatomical heterogeneity in patients, they serve as reasonable approximation. A clear recommendation on imaging settings cannot be given at this point as the optimization is highly patient-specific. Such patient-specific imaging parameter optimization could be based on personalized phantoms or simulated X-ray images (e.g., from a pre-treatment computed tomography) [57], [58]. Treatment duration per fraction, largely influenced by the fractionation scheme and the use of flattening filter free (FFF) beams, may also impact the selection of imaging settings.

A study limitation was that the used imaging geometry may not reflect all gantry angles employed during VMAT delivery. Furthermore, no treatment beam, which could degrade image quality through cross-scattering, was considered during X-ray imaging [59], [60], [61], [62]. Due to beam proximity, simultaneous MV irradiation was expected to have a larger impact on gantry-mounted systems. Preliminary assessments with a 5 × 5 6 MV FFF field revealed a CNR decrease of 0.3 ± 1.3% for ETD and 2.3 ± 3.0% for XVI (Supplementary Section B). However, the influence during patient treatment is highly application-specific and requires further investigations.

Markerless intra-fractional lung tumor motion monitoring at C-arm LINACs has shown promising results using gantry-mounted kV imaging devices, [12], [15], [25], [26], [33], [34], but has not yet become a widely established clinical workflow [14]. This work extended experimental feasibility studies by exploring a room-mounted system. Addressing patient-specific imaging settings, feasible patient cohorts, lesion sizes, motion management techniques or tumor localization methods, is a topic of future research.

In summary, the performance of the room-mounted imaging device investigated in this study aligned with the gantry-mounted system with respect to CNR and air kerma (considering default imaging settings and average beam-on times) in two anthropomorphic phantoms, one mimicking a real patient. The room-mounted system showed a preferable cool-down behavior. However, default imaging settings should be optimized to reduce the imaging dose for patients and extend fluoroscopy durations while maintaining image quality.

## CRediT authorship contribution statement

**Hannah Jungreuthmayer:** Writing – review & editing, Writing – original draft, Visualization, Software, Methodology, Investigation. **Barbara Knäusl:** Writing – review & editing, Writing – original draft, Supervision, Project administration, Funding acquisition, Conceptualization. **Julius Arnold:** Writing – review & editing, Methodology, Investigation. **Martin Buschmann:** Writing – review & editing, Investigation. **Andreas Renner:** Writing – review & editing, Conceptualization. **Maximilian Schmid:** Writing – review & editing, Funding acquisition. **Dietmar Georg:** Writing – review & editing, Resources. **Wolfgang Lechner:** Writing – review & editing, Writing – original draft, Supervision, Conceptualization.

## Funding

The financial support by the Austrian Federal Ministry of Economy, Energy and Tourism, the National Foundation for Research, Technology and Development and the Christian Doppler Research Association is gratefully acknowledged.

## Declaration of competing interest

The authors declare the following financial interests/personal relationships which may be considered as potential competing interests: Within the frame of the Christian Doppler Research Association this study was financially supported by Brainlab SE and Elekta AB. Martin Buschmann received travel and speaker fees from Brainlab. Given their role as Editor-in-Chief, Barbara Knäusl had no involvement in the peer-review of this article and had no access to information regarding its peer-review. Full responsibility for the editorial process for this article was delegated to another journal editor.

## Appendix A Supplementary data

MMC S1 Extended technical specifications, experimental details and figures supporting the reported results. MMC S1
