# Supplementary material for: Assessing the technical capability of a room- and a gantry-mounted kV imaging device for intra-fractional fluoroscopy during stereotactic lung radiotherapy at a C-arm linear accelerator
Source: Phys Imaging Radiat Oncol. 2026 May 2;39:100988. doi: 10.1016/j.phro.2026.100988 (PMC13157211; doi:10.1016/j.phro.2026.100988)
Supplement: MMC S1 — Extended technical specifications, experimental details and figures supporting the reported results. [file mmc1.pdf]

Supplementary material

A. Technical specifications

ExacTrac Dynamic (ETD) (Brainlab, Munich, Germany): Two X-ray sources are located in the floor with their detector mounted diagonally from the tube on the ceiling. Source-axis distance and source-detector distance are 2100 mm and 3500 mm, respectively . The clinical ETD provides a field-of-view (FOV) at the isocenter of 18 cm × 18 cm, a detector area of 29.8 cm × 29.8 cm and images with pixel dimensions of 768 × 768 [38]. The prototype software saves images with pixel dimensions of 1536 × 1536, therefore 2x2 software binning was applied to the prototype images to match clinical image dimensions.

XVI (Elekta AB, Stockholm, Sweden): XVI is a gantry-mounted imaging system, with a source-axis distance of 1000 mm and source-detector distance of 1536 mm. It has a FOV at the isocenter of 27.67 cm × 27.67 cm (with the S20 collimator), a detector area of 40.96 cm × 40.96 cm and produces clinical XVI images with pixel dimensions of 512 × 512 [39].

NOMEX Multimeter (PTW, Freiburg, Germany): The semiconductor detector has a dose measurement range of 50 nGy ··· 500 Gy [42].

CIRS Dynamic phantom (CIRS, Norfolk, USA): The phantom approximates the human body in structure and size using simplified geometries. It contains a lung, a lung tumor and a spine with cortical and trabecular bone, which were constructed using tissue equivalent epoxy materials. Their linear attenuation is within 1% of actual attenuation for bone and water and within 3% for lung. The electron densities for plastic water, lung, cortical bone, trabecular bone and soft tissue target are 3.35×10^{23}, 0.69×10^{23}, 5.95×10^{23}, 3.86×10^{23} and $3.43\times10^{23}\text{cm}^{-3}$ [43]. The phantom body is connected to an actuator box enabling arbitrary three-dimensional tumor motion trajectories.

LUNGMAN: The phantom approximates shape, radiation absorption and HU number of a human body. It contains the following anatomical landmarks: rib cage, thoracic spine, sternum, clavicles, detailed bronchovascular tree, mediastinum, diaphragm and different lung tumor inserts. Soft tissue consists of polyurethane and the bones are made of epoxy resin and calcium carbonate [44].

B. Influence of MV scatter

To estimate the influence of MV scatter on the CNR, a 5x5 6 MV FFF beam was irradiated while acquiring four fluoroscopy frames with default settings (Table 1) using the CIRS phantom for ETD and XVI. For reference, the experiments were repeated on the same day without an MV beam. The measurement setup and CNR evaluation were identical to Section 2.2 and 2.3. There was a mean decrease in CNR of 0.3±1.3% for ETD, while it decreased by 2.3±3.0% for XVI (Figure S1). Uncertainties were calculated by applying Gaussian error propagation.

C. Supporting figures and tables

No.	Quantity or source of uncertainty	Uncertainty type	probability density function	rel. standard uncertainty
				%
1	Reproducibility range ETD	A	Normal	1.9–8.0
2	Reproducibility range XVI	A	Normal	4.3–9.1
3	Calibration	B	Normal	1.3

Table S1: Uncertainty budget of the entrance air kerma measurements with the NOMEX Multimeter (coverage factor k=1) [42]. Reproducibility was measured, while calibration was taken from the calibration certificate of the NOMEX Multimeter.

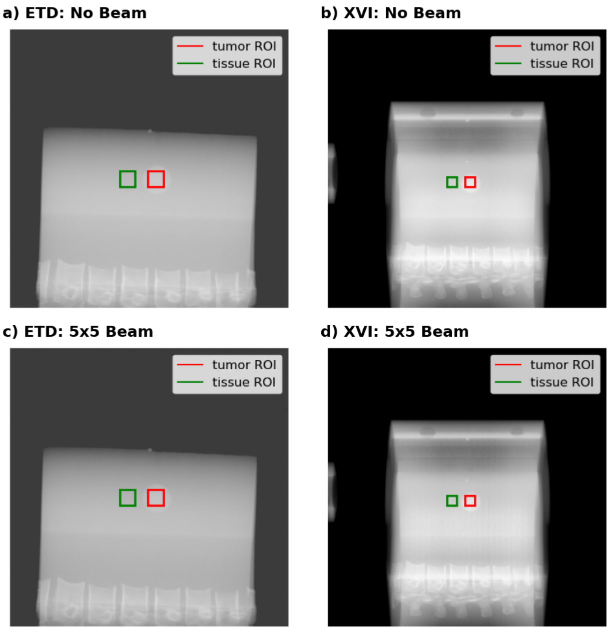

Figure S1: a) and b) show an ETD prototype and an XVI CIRS phantom image (default settings) acquired without MV irradiation. c) and d) show the same with an MV beam. ROIs in the tumor and surrounding tissue are indicated.

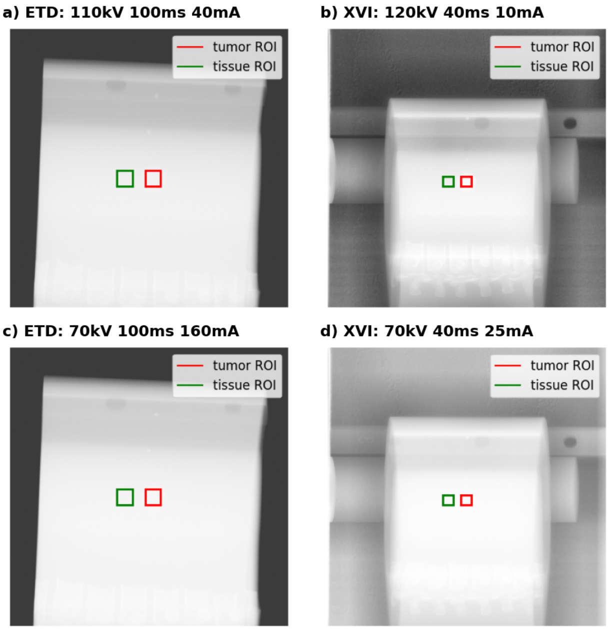

Figure S2: a) and b) show an ETD prototype and an XVI CIRS phantom image acquired at the respective default tube voltage with a lower than default tube current for illustrative purposes. c) and d) show the same at the respective default tube current with a lower than default tube voltage. ROIs in the tumor and surrounding tissue are indicated.

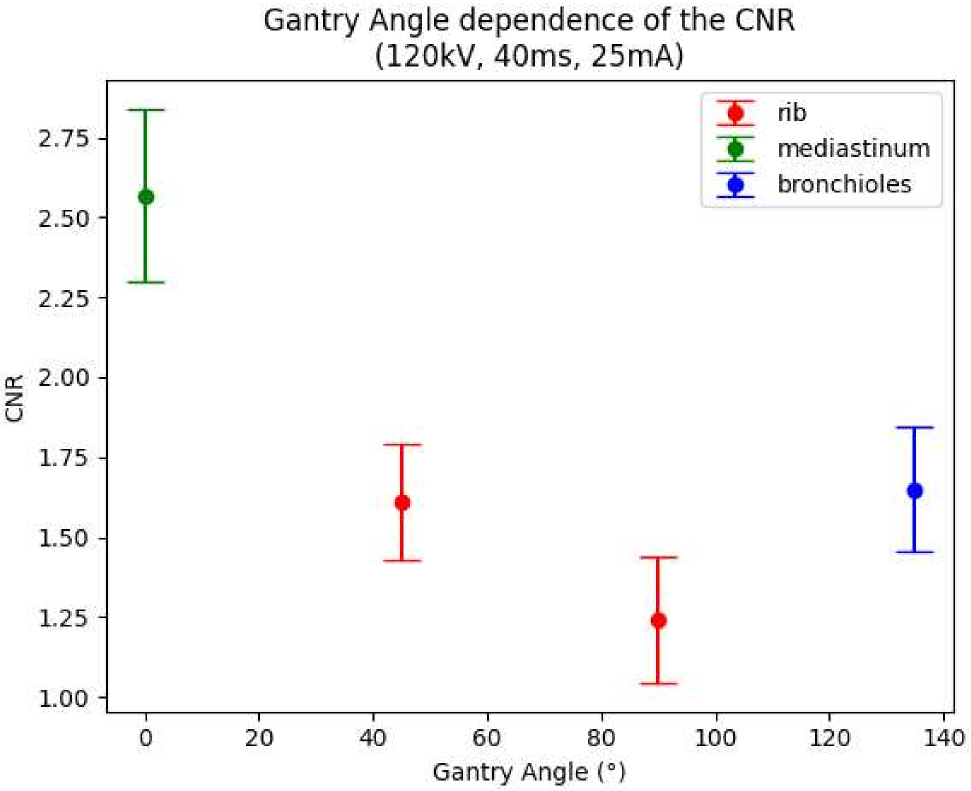

Figure S3: CNR between LUNGMAN *tumor 2* and tissue ROI as a function of the gantry angle for 120 kV, 40 ms and 25 mA (default setting). The respective tissues in the background are listed in the legend.

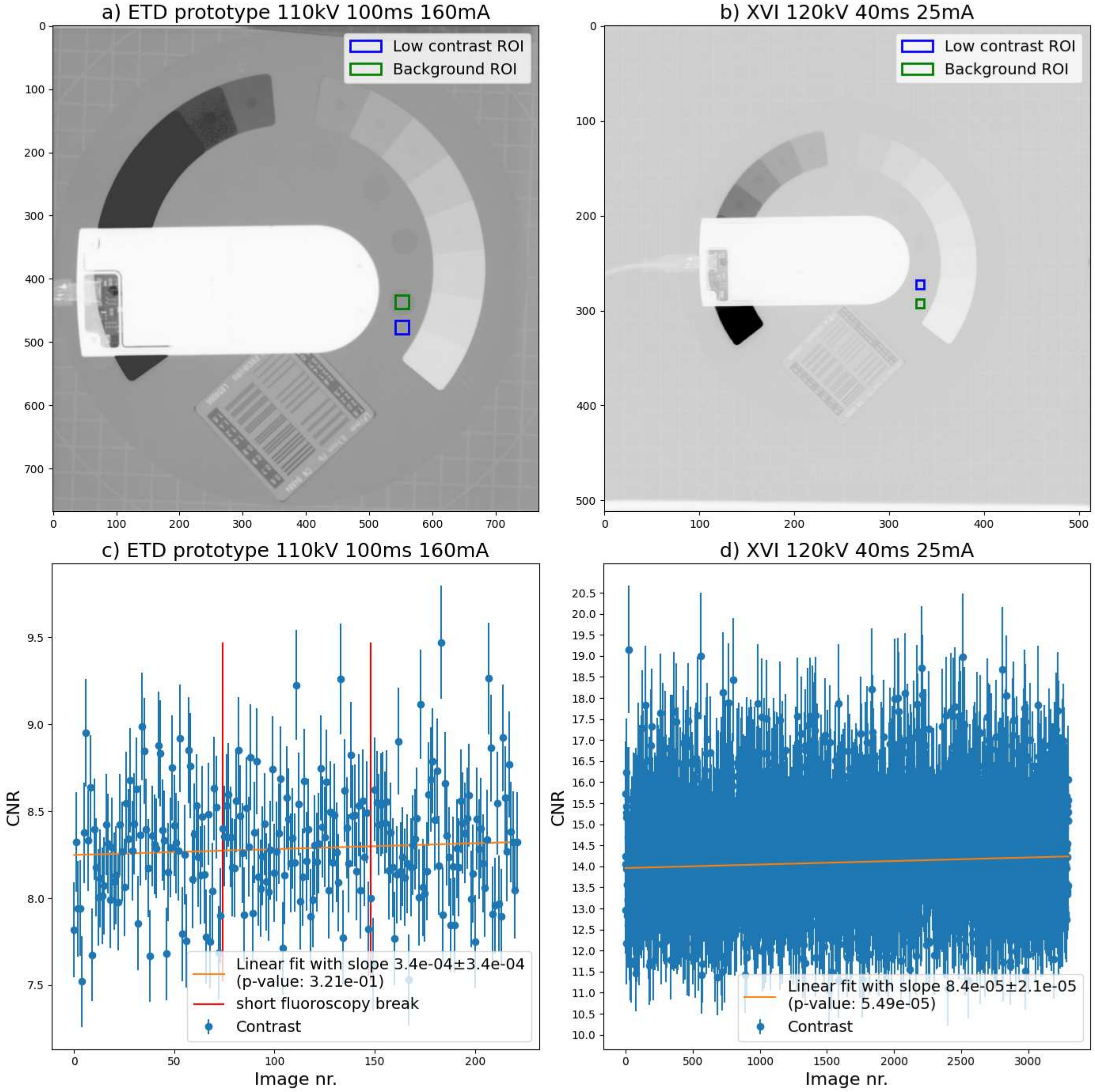

Figure S4: a-b) PTW NORMI RAD/FLU phantom with indicated ROIs in the background and a low contrast region. For ETD a copper plate was positioned on the phantom to avoid detector saturation. c-d) CNR between the ROIs as a function of time (actually image number). 74x3 ETD fluoroscopy frames were acquired with the default lung imaging setting. The vertical red lines indicate short breaks between subsequent fluoroscopy sequences (74 frames each). For XVI, 3300 frames were acquired with the default lung imaging setting.

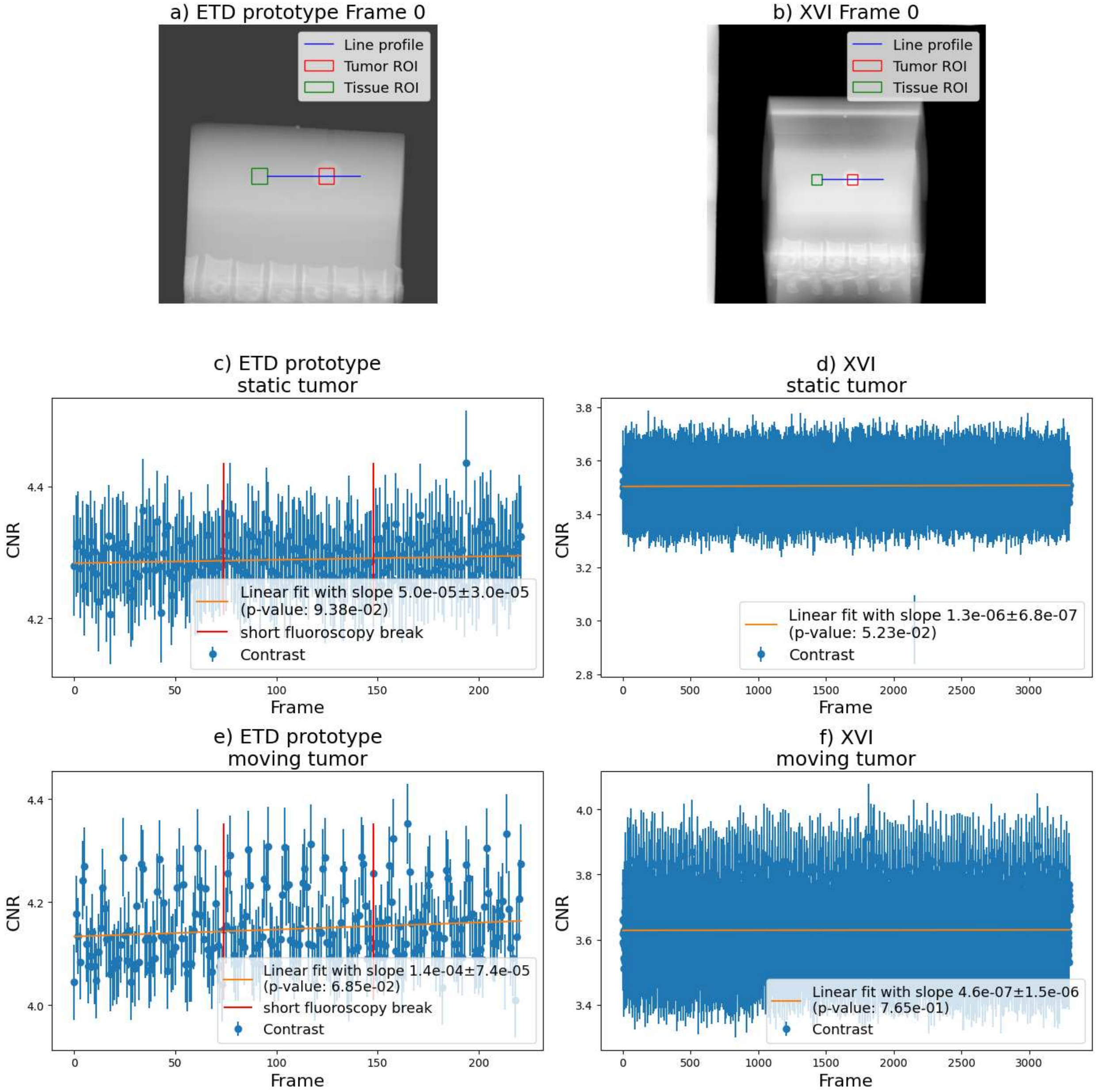

Figure S5: a-b) CIRS phantom with indicated ROIs in the tumor and the tissue, as well as a line profile. c-d) CNR between the ROIs as a function of time (actually image number) for a static tumor. 74x3 ETD fluoroscopy frames were acquired, with the vertical red lines indicating short breaks between subsequent fluoroscopy sequences (74 frames each). For XVI, 3300 frames were acquired. e-f) same as c-d) for the moving tumor. The tumor ROI position for CNR calculation was determined by analyzing a line profile across the tumor, the tissue ROI remained static and outside of the tumor motion range.

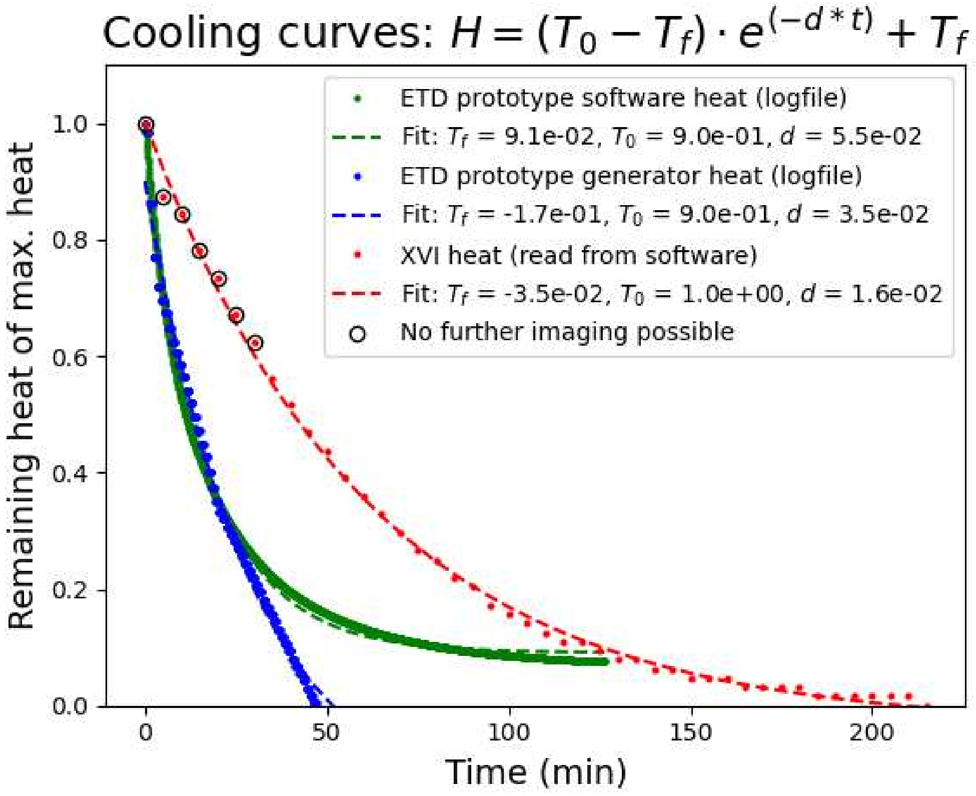

Figure S6: ETD and XVI cooling curves after reaching a fluoroscopy endpoint. The blue and the green curve indicate ETD prototype cooling curves after no further fluoroscopy sequence with the maximum frame number was possible. Blue indicates the logged generator-reported heat and green indicates the logged heat as approximated by the ETD prototype software. The red curve shows the XVI cooling curve after reaching a point where no further imaging was possible at all. Black circles around the red data points indicate that still no further imaging was possible. The curves were fitted with an exponential function $H = (T_0 - T_f) \cdot exp(-d \cdot t) + T_f$, where H is the percentage of the maximum heat that was reached, T_0 is the maximum heat percentage (in case of an ideal exponential decay this should be 1, i.e. 100%), T_f is the final heat percentage and d is a decay constant.
